# Supplementary material for: A qualitative meta-synthesis of service users’ and carers’ experiences of assessment and involuntary hospital admissions under mental health legislations: a five-year update
Source: BMC Psychiatry. 2024 Jun 27;24:476. doi: 10.1186/s12888-024-05914-w (PMC11209989; doi:10.1186/s12888-024-05914-w)
Supplement: Supplementary file 1 — Supplementary Material 1 [file 12888_2024_5914_MOESM1_ESM.docx]

# Supplementary information

A qualitative meta-synthesis of service users’ and carers’ experiences of assessment and involuntary admissions under mental health legislations: a five-year update

##

Search strategy 1 March 2023

Medline (OvidSP):

1. service-user* or patient* or consumer* or carer* or famil* or caregiver* or caregivers/ or relative* or inpatient* or client* or ((lived or life) adj experience*) or survivor*
2. "mental health act" or section* or 'mental treatment act' or ((compuls* or involuntar* or coer* or forced or detention or detained or refusal or mandat* or civil or appeal* or advoc*) adj2 (hospital* or admiss* or admit* or readmiss* or commit* or assess* or treat* or healthcare))
3. mental disorders/ or ((mental* or psychologic* or psychiatr*) adj2 (health or disorder* or disease* or deficien* or illness* or problem*)).ti,ab,sh.
4. qualitative research/ or interview/ or qualitative or (theme$ or thematic) or 'ethnological research' or (humanistic or existential or experiential or paradigm$) or (field adj (study or studies or research)) or ((purpos$ adj4 sampl$) or (focus adj group$)) or 'observational method$' or 'content analysis' or ((discourse$ or discurs$) adj3 analys?s) or 'narrative analys?s' or (grounded adj (theor$ or analys?s)) or 'action research' or (account or accounts or unstructured or openended or open ended or narrative$) or (lived adj experience$)
5. 1 and 2 and 3 and 4
6. limit 5 to yr=“2018-Current”

Embase (OvidSP):

1. service-user* or patient* or consumer* or carer* or famil* or caregiver* or caregiver/ or relative* or inpatient* or client* or ((lived or life) adj experience*) or survivor*)
2. "mental health act" or section* or 'mental treatment act' or ((compuls* or involuntar* or coer* or forced or detention or detained or refusal or mandat* or civil or appeal* or advoc*) adj2 (hospital* or admiss* or admit* or readmiss* or commit* or assess* or treat* or healthcare))
3. mental disease/ or ((mental* or psychologic* or psychiatr*) adj2 (health or disorder* or disease* or deficien* or illness* or problem*)).ti,ab,sh.
4. qualitative research/ or interview/ or qualitative or (theme$ or thematic) or 'ethnological research' or (humanistic or existential or experiential or paradigm$) or (field adj (study or studies or research)) or ((purpos$ adj4 sampl$) or (focus adj group$)) or 'observational method$' or 'content analysis ' or ((discourse$ or discurs$) adj3 analys?s) or 'narrative analys?s ' or (grounded adj (theor$ or analys?s)) or 'action research' or (account or accounts or unstructured or openended or open ended or narrative$) or (lived adj experience$)
5. 1 and 2 and 3 and 4
6. limit 5 to yr=“2018-Current”

PsycINFO (OvidSP):

1. service-user* or patient* or consumer* or carer* or famil* or caregiver* or caregivers/ or relative* or inpatient* or client* or ((lived or life) adj experience*) or survivor*
2. "mental health act" or section* or 'mental treatment act' or ((compuls* or involuntar* or coer* or forced or detention or detained or refusal or mandat* or civil or appeal* or advoc*) adj2 (hospital* or admiss* or admit* or readmiss* or commit* or assess* or treat* or healthcare))
3. mental disorders/ or ((mental* or psychologic* or psychiatr*) adj2 (health or disorder* or disease* or deficien* or illness* or problem*)).ti,ab,sh.
4. qualitative research/ or interview/ or qualitative or (theme$ or thematic) or 'ethnological research' or (humanistic or existential or experiential or paradigm$) or (field adj (study or studies or research)) or ((purpos$ adj4 sampl$) or (focus adj group$)) or 'observational method$' or 'content analysis' or ((discourse$ or discurs$) adj3 analys?s) or 'narrative analys?s' or (grounded adj (theor$ or analys?s)) or 'action research' or (account or accounts or unstructured or openended or open ended or narrative$) or (lived adj experience$)
5. 1 and 2 and 3 and 4
6. limit 5 to yr=“2018-Current”

HMIC (OvidSP):

1. service-user* or patient* or consumer* or carer* or famil* or caregiver* or carers/ or relative* or inpatient* or client* or ((lived or life) adj experience*) or survivor*
2. "mental health act" or section* or 'mental treatment act' or ((compuls* or involuntar* or coer* or forced or detention or detained or refusal or mandat* or civil or appeal* or advoc*) adj2 (hospital* or admiss* or admit* or readmiss* or commit* or assess* or treat* or healthcare))
3. mental disorders/ or ((mental* or psychologic* or psychiatr*) adj2 (health or disorder* or disease* or deficien* or illness* or problem*)).ti,ab,sh.
4. qualitative research/ or interviews/ or qualitative or (theme$ or thematic) or 'ethnological research' or (humanistic or existential or experiential or paradigm$) or (field adj (study or studies or research)) or ((purpos$ adj4 sampl$) or (focus adj group$)) or 'observational method$' or 'content analysis' or ((discourse$ or discurs$) adj3 analys?s) or 'narrative analys?s' or (grounded adj (theor$ or analys?s)) or 'action research' or (account or accounts or unstructured or openended or open ended or narrative$) or (lived adj experience$)
5. 1 and 2 and 3 and 4
6. limit 5 to yr=“2018-Current”

Social Science Citation Index (Web of Science):

1. service-user* or patient* or consumer* or carer* or famil* or caregiver* or relative* or inpatient* or client* or ((lived or life) N0 experience*) or survivor*
2. "mental health act" or section* or "mental treatment act" or ((compuls* or involuntar* or coer* or forced or detention or detained or refusal or mandat* or civil or appeal* or advoc*) N2 (hospital* or admiss* or admit* or readmiss* or commit* or assess* or treat* or healthcare))
3. mental disorders or ((mental* or psychologic* or psychiatr*) N2 (health or disorder* or disease* or deficien* or illness* or problem*))
4. qualitative research or interview or qualitative or (theme* or thematic) or "ethnological research" or (humanistic or existential or experiential or paradigm$) or (field N0 (study or studies or research)) or ((purpos* N4 sampl*) or (focus N0 group*)) or "observational method*" or "content analysis" or ((discourse* or discurs*) N3 analys?s) or "narrative analys?s" or (grounded N0 (theor* or analys?s)) or "action research" or (account or accounts or unstructured or openended or "open ended" or narrative*) or (lived N0 experience*)
5. 1 and 2 and 3 and 4
6. limit from 01/01/2018-01/03/2023
